# The Future of Stroke Interventions

**DOI:** 10.5041/RMMJ.10404

**Published:** 2020-04-29

**Authors:** Eitan Abergel

**Affiliations:** Invasive Neuroradiology Unit, Rambam Health Care Campus, Haifa, Israel

**Keywords:** Digital health, endovascular robotics, large-vessel occlusion, neuroprotection

## Abstract

Mechanical thrombectomy (MT) has revolutionized the treatment of large-vessel occlusion stroke and markedly improved patient outcomes. Unfortunately, there remains a large proportion of patients that do not benefit from this technology. This review takes a look at recent and upcoming technologies that may help to increase the number of MT-treated patients, thereby improving their outcomes. To that end, an overview of digital health solutions, innovative pharmacological treatment, and futuristic robotic endovascular interventions is provided.

## INTRODUCTION

Stroke is a major burden in Western society, is the most frequent cause of acquired disability, and the fifth most frequent cause of mortality in the United States of America (USA).

In 2015 five major randomized controlled trials were published^1^–^5^ that revolutionized the management, treatment, and outcome of stroke due to large-vessel occlusion (LVO). Grouping those five studies, the Hermes meta-analysis^6^ demonstrates that mechanical thrombectomy (MT) plus intravenous thrombolysis (IVtPa), versus IVtPa alone, results in a 50% relative risk reduction of significant neurological disability, with an outstanding number of 4 needed to treat to avoid 1 severe neurological deficit.

Shortly after those publications, the American Heart Association (AHA)^7^ and the European Stroke Organisation (ESO)^8^ guidelines embraced endovascular therapy for LVO stroke treatment, and there is now no doubt that MT dramatically improves the outcomes of LVO patients.

To prove clinical efficacy, initial MT studies focused on a limited subset of patients for whom the procedure was likely to be highly beneficial; this was necessary after the failure of previous inadequately designed studies.^9^ Today the neurovascular community is progressively pushing the boundaries of MT, proposing the procedure to a broader group of patients by enlarging the treatment time window,^10^,^11^ including patients with large infarct volumes,^12^ older patients,^13^ and more distal occlusion sites.^14^

Nevertheless, not all eligible patients benefit from this therapy. Some challenges remain to be overcome in order to deliver this advanced therapy to the most widespread proportion of LVO stroke patients.

The patient pathway from symptoms onset up to MT is complex, involving many steps and actors. Medical teams must act efficiently to bring the right patient to the angiography suite within the shortest time frame. At each step, precious time can be lost, resulting in a potentially worse clinical outcome, or even preventing patients from undergoing MT at all.

This review exposes some of those difficulties and will give insights into potential future solutions.

## THE PREHOSPITAL CHALLENGE

Out of hospital, accurate early identification of patients requiring MT is a significant challenge. Medical professionals must keep in mind that only patients with LVO and salvageable brain are candidates for endovascular treatment; such patients represent around 10%–15% of all stroke patients.^15^,^16^

In contrast to myocardial infarction, in which a simple electrocardiogram is sufficient to determine which patient needs an urgent primary coronary intervention (PCI), in stroke, brain imaging is mandatory before referring a patient for MT. Therefore, when stroke is suspected, medical staff in the field must already decide whether or not they should bring the patient to the nearest primary stroke center (PSC), where the patient can undergo rapid clinical and imaging triage. Only then, if the patient is found to be eligible, will he or she be transferred secondarily to a comprehensive stroke center (CSC) for MT. Alternatively, should every patient be sent directly to the CSC in which MT is available? This could carry a high cost, depending on the patient’s geographical location, of longer transportation times.

The first approach prevents futile transfers to the CSC and decreases the door-to-needle time for IVtPa. The second approach would significantly reduce door-to-groin puncture time for LVO patients, but would benefit less than 10%–20% of all stroke patients,^15^ resulting in an overwhelming overload of non-LVO patients on the CSC emergency room, preventing or delaying IVtPa administration, and causing delays in the management of non-stroke patients (so-called stroke mimics).

The above dilemma reveals the critical need for a prehospital tool to identify MT candidates. In the absence of a “brain electrocardiogram,” to correctly select LVO patients, clinical prehospital scoring systems are utilized. Observational data have revealed that the more severe the stroke, the greater the likelihood of experiencing an LVO. Several simplified clinical scales have been studied for this purpose, but none of them achieved both high sensitivity and specificity,^17^ and to date there is no clear evidence of their value for managing dispatch rules in the field.

The RaceCat^18^ trial by Ribo and colleagues is an ongoing randomized clinical trial that assesses this particular question using the RACE scale (Rapid Arterial oCclusion Evaluation; a five-item, nine-point, simplified stroke scale) in the province of Barcelona in Catalonia, Spain. Intermediate results show that, for the most severe clinical presentation (score of 9/9), 60% of patients eventually require MT. However, as the score goes down, the prediction rate of the need for MT drops to 30%.

For the many patients suffering moderate to severe strokes, using the full National Institutes of Health Stroke Scale/Score (NIHSS) and DynaCt (flat panel computed tomography [CT] performed in the catheterization laboratory) can increase the prediction rate for LVO (and the need for MT) back to 60%. However, the full NIHSS seems to be cumbersome and too complicated for use in the prehospital setup: it is time-consuming, and its accuracy is highly related to the level of experience and training of the caregiver in the field.^17^

In an effort to create a fast, homogeneous, and reliable scoring methodology, machine-based software is under development that utilizes artificial intelligence and deep-learning. For example, CVAid® (CVAid Medical, Tel Aviv, Israel) is a portable application that uses a cellphone camera and microphone to automatically generate an NIHSS; unpublished initial results seem promising, showing good correlation to neurologist-performed NIHSS evaluation. Validation studies are still required, and further trials should determine its efficacy in a clinical setup.

However, even accurate clinical scoring is not enough; the destination decision for patient transfers must be integrated with many other variables such as eligibility for IVtPa, online transportation time differences between CSC and PSC, and availability of human and material resources. Apps like Join® (Allm Inc., Tokyo, Japan) or Pulsara® (Pulsara, Bozeman, MT, USA) simplify the sharing of clinical and radiological medical information and facilitate effective communication, offering a wide range of usages, including messaging, group chatting, pictures archiving and communication system (PACS) integration, streaming of live feed videos, global positioning system (GPS) real-time guidance, and time tracking. These features can significantly streamline patient workflow so that stroke team members can efficiently coordinate the patient pathway. In their single-center study Reeves and co-workers compared door-to-needle IVtPa times with and without use of the Pulsara® application.^19^ Their data showed that the app allowed an absolute 40-minute reduction in door-to-needle IVtPa time (from 87 to 47 minutes), which represents a 46% relative time reduction from the cases not using the application.

Once patients arrive at a medical center, urgent imaging, either by computed tomography or magnetic resonance, is performed and must be promptly analyzed by qualified radiologists to determine if MT is required. However, image interpretation is subject to inconsistent local expertise, time delays, and varies between institutions. Even at experienced facilities, activation of interhospital communication for LVO triage and transport to a thrombectomy center can be operationally challenging.

To address this need, artificial intelligence tools using deep machine learning algorithms are being developed, which can: (1) automatically generate a patient Alberta Stroke Program Early CT Score (ASPECTS) (radiological assessment of the middle cerebral artery [MCA] territory infarct size, calculated on non-contrast CT); (2) create perfusion maps and determine perfusion mismatch; and (3) detect LVO. These data are then automatically pushed, with notifications, to the relevant physician and team at the hub center on a cellphone application, thereby allowing rapid decision-making and quick instruction transfer to the hospital. Over the past 5–6 years, several software platforms have been commercialized, of which the most prominent are: RapidAI™ (Medtronic RapidAI, Menlo Park, CA, USA), VIZ.ai® (Viz.ai, Tel Aviv, Israel), and e-ASPECTS (Brainomix Ltd, Oxford, UK). These applications share the same concept but have variations regarding the algorithm and available features.

Initial clinical data focus mainly on accuracy and compare artificial intelligence (AI) performance and precision to the interpretation of experienced radiologists.

The relative accuracy of the automatic ASPECTS to humans is similar or even better. Albers et al. evaluated the performance of automatically generated RapidAI™ASPECTS relative to scores determined by experienced radiologists.^20^ The non-contrast CTs of stroke patients were assessed and compared to a matched diffusion-weighted imaging (DWI). The RapidAI® software was found to be more accurate than experienced clinicians in identifying early evidence of brain ischemia as documented by DWI. Similar results were achieved using the Brainomix e-ASPECTS software.^21^

Amukotuwa et al. used the RapidAI^™^ software in an unselected population undergoing computed tomography angiography (CTA) for suspected stroke.^22^ With regard to LVO detection, they found that the software’s algorithms were oriented for higher sensitivity at the price of lower specificity. They also found a sensitivity, negative predictive value, and specificity of 0.94, 0.98, and 0.76, respectively, for intracranial LVO detection.^22^ The authors concluded that the “algorithm could be used in the emergent setting as a screening tool to alert radiologists and expedite formal diagnosis.”^22^^(p2790)^

Future data should evaluate the clinical benefit of those tools. To our knowledge, there is no such published data. However, a poster was presented during the American Heart Association International Stroke Conference 2019 on the implementation of Viz.ai® for automatic LVO detection, indicating a 52-minute mean reduction time from imaging to decision-making.^23^ These unpublished data need further confirmation in upcoming trials.

Future applications that integrate all the above-mentioned software platforms will undoubtedly play a key role in stroke networking and in helping care-givers to rapidly and efficiently identify potential candidates for MT, as well as in providing a communication platform and optimized workflow, and will hopefully reduce the treatment time frame, resulting in improved patient outcomes.

## “ENLARGING” THE TIME WINDOW

Stroke is and will remain a highly time-sensitive pathology. Despite all efforts to streamline work-flow, some patients might still experience unfavorable outcomes due to either a rapidly growing infarction resulting from poor collateral circulation (“fast progressors” profile) or due to very long patient transportation times to a CSC/PSC for any number of reasons.

An ideal tool for stroke, described by some as the “holy grail,” would be a neuroprotective drug that could preserve brain cells in the penumbra until the brain heals, or extend the window of time to preserve neurons in stroke patients until reperfusion is achieved either by IVtPa or MT.

Glutamate is a ubiquitous excitatory neurotransmitter in the brain. In his 1970 publication, J.W. Olney showed that high extracellular levels of glutamate act as a neurotoxin in mice, by flooding neurons with calcium, triggering a downstream neurotoxic cascade and generating intracellular free radicals, resulting in enzyme activation and eventually apoptosis.^24^ This excitotoxicity mechanism is believed to be a culprit in a range of neurological diseases, including stroke. In this same publication, Olney showed that glutamate plays a key role in ischemic brain damage and that drugs which decrease the accumulation of glutamate or block its postsynaptic effects may be a rational therapy for stroke, giving the medical community hope for effective stroke therapy.

Following those results and through to 2003, over 1,000 potential neuroprotective drugs have been tested in over 8,516 studies^25^—all of which have failed. To help research and improve future studies, design, and results, the Stroke Treatment Academic Industry Roundtable (STAIR) committee was established to produce guidelines and recommendations for researchers.^26^

The pharmaceutical agent NXY-059 traps free radicals. It has neuron-protective effects in animal stroke models. Published In 2006 using the STAIR recommendations, the SAINT I study was a double-blind, placebo-controlled trial involving 1,722 acute ischemic stroke patients who were randomly assigned to receive a placebo infusion or intravenous NXY-059 within 6 hours of stroke onset; the study showed, for the first time, a modest but statistically significant clinical benefit over placebo.^27^ Subsequently, a similar, more extensive clinical trial, SAINT II, was conducted. However, despite high hopes, the study failed to confirm the initially encouraging results,^28^ and the authors concluded that “NXY-059 is ineffective for the treatment of acute ischemic stroke within 6 hours after the onset of symptoms.”^28^^(p562)^

Following the disappointing initial results of the SAINT II and many other studies, researchers and industry seemed to doubt the potential for neuroprotective drugs to reduce stroke damage.

Nevertheless, in 1998, research had already indicated that neurotoxicity was triggered by calcium influx through glutamate NMDA receptors (NMDAr), and that the level of calcium influx alone was not the only determinant of cell survival or death, rather, it was actually the calcium pathway,^29^ known as the “source specificity hypothesis.” In a 1999 publication, Tymianski and colleagues showed that PSD-95, which is part of the scaffolding synaptic proteins, binds to the NMDAr and intracellular neuronal nitric oxide synthase (nNOS) and that, in the presence of intracellular calcium, PSD-95 plays a crucial role in the mechanism by which NMDAr activity triggers nitric oxide production by nNOS and excitotoxicity.^30^

After understanding the mechanism and role of PSD-95, a specific inhibitor was developed. The inhibitor, NA-1, is an interfering peptide that disrupts the NMDAr–PSD95–nNOS complex and dissociates NMDArs from downstream neurotoxic signaling, without blocking the normal synaptic function of NMDArs or calcium influx.^31^ Hence, NA-1 seems to be the ideal candidate for neuroprotection in stroke. However, why should NA-1 perform better than more than 1,000 other drugs that have been tested?

As with MT randomized trials, the early experience was disappointing. Following the failed IMS III study,^9^ it took neurointerventionalists, proper patient selection, and advances in technology to pursue the MR Clean trial,^32^ which eventually established the benefit of MT for acute ischemic stroke.

Neuroprotective trials have suffered the same flaw of the MT studies, i.e. improper patient selection and suboptimal trial design. However, NA-1 studies, as MR Clean did, seems to have chosen the right study design and optimal patient selection, and hopefully will prove the concept of neuroprotection. Extensive preclinical data exist for NA-1, including several studies in non-human primates, using a MCA occlusion (MCAO)-reperfusion model that reproduces LVO stroke followed by MT reperfusion. Cynomolgus macaques treated with NA-1 3 hours after MCAO showed a significant reduction in infarct size on DWI, which was confirmed by histology. Furthermore, treated animals had better clinical outcomes, as shown by the non-human primate stroke scale (NHPSS).^33^

A phase 2, multicenter, double-blind, randomized controlled clinical trial, ENACT (Evaluating Neuroprotection in Aneurysm Coiling Therapy), published in 2012, examined NA-1 efficacy and safety in patients undergoing endovascular aneurysm embolization, an intervention known to produce mini-strokes detectable on post-procedural MRI. The treated group exhibited a lower number of DWI lesions and improved clinical outcomes, providing evidence that effective pharmacological neuroprotection in humans is possible.^34^

## ONGOING TRIALS

There are two ongoing large-scale acute stroke phase 3 clinical trials: FRONTIER and ESCAPE NA-1.

The FRONTIER (Field Randomization of NA-1 Therapy in Early Responders) trial is a prehospital trial in which paramedics randomly administer NA-1 to suspected stroke patients in the first 3 hours of symptoms onset. The study will determine the efficacy and safety of NA-1 in reducing global disability in patients with acute stroke, including minor and major stroke. The study’s estimated completion date is May 2021.

Focusing on LVO stroke, the ESCAPE NA-1 trial is including patients selected for endovascular therapy who have a limited necrotic core (ASPECTS score>6) and good collaterals. Enrolled patients are randomized to receive either NA-1 or placebo. Trial design is particularly interesting as it focuses on eligible patients who undergo mechanical reperfusion. Indeed, administering a neuroprotective drug, as efficient as it can be, makes no sense if there is no reperfusion at some point. The ESCAPE NA-1 trial is testing, for the first time, a neuroprotective drug in an occlusion-reperfusion scenario in the era of MT. Results for this trial were first published on February 20, 2020.^35^ Overall, the results seem to be disappointing, with no clinical benefit for the neuroprotective drug over placebo.^35^ However, looking at the subgroup of patients who underwent MT without receiving IVtPa, there is a clear, statistically significant benefit of NA-1. The results are even quite impressive, including an 11% absolute increase of the primary end point (modified Rankin score 0–2), reduction in infarct volume, and, most interestingly, a 7.5% absolute reduction of mortality. The absence of benefit for patients receiving IVtPa is likely to be due to enzymatic cleavage of NA-1 by plasmin, leading to subtherapeutic concentrations of the study drug. This pharmacokinetic phenomenon was unknown to the study investigators before the trial, explaining the inclusion of thrombolysed patients.

A new study, excluding IVtPa patients, is required. Nevertheless, there is now an unambiguous indication that neuroprotection in stroke is feasible and probably very efficient. If the “no-IVtPa” subgroup results are proven to be accurate, neuroprotective drugs could represent the next revolution in acute stroke management.

## THE GEOGRAPHICAL CHALLENGE

Endovascular capable stroke centers represent roughly one-third of all stroke centers in the USA (327 of 1,148) and in France (37 of 132). In Europe, the number of endovascular therapy capable centers varies from 135 in Germany (1.7 per million inhabitants) to 28 in the United Kingdom (0.1 per million inhabitants).^36^ Estimations indicate that even with a direct transfer approach, only 56% of Americans are within 60 min ground transfer proximity to a CSC, and more than 20% are, at best, between 3 to 6 hours away.^37^ As a result, even in a streamlined workflow, in some settings, it is more difficult to transport patients directly to a CSC.

Population distribution and density are essential considerations when planning stroke services. Opening new, strategically placed CSCs is essential in some regions, but it might be impossible to achieve full territory coverage.

Telestroke is one of the most successful telemedicine applications and has been in use for more than two decades. Using telestroke, rural centers can effectively treat ischemic stroke patients with intravenous IVtPa on-site rather than transferring them to the closest available stroke center for delayed evaluation and treatment, often outside thrombolytic time windows. Studies have shown that tele-stroke can increase the rate of IVtPa, reduce door-to-needle time, and improve patient outcomes.^38^,^39^

Will it be possible for telemedicine hub centers to also offer remote endovascular procedures?

Corindus® (Waltham, MA, USA), founded in 2002 by Professor Rafael Beyar and recently sold to Siemens®, is a precision vascular robotics company. Their CoroPath robotic-assisted system can be mounted on the catheterization laboratory bed. It was originally developed with the goal of reducing radiation exposure to the operator and his team. The physician performs the procedure sitting behind a shielded interventional cockpit located in, or outside of, the angiography suite.

The first-generation robot, CoroPath 200, allows for control of a guidewire and one balloon or stent. In the PRECISE trial, 164 patients were enrolled in a multicenter, non-randomized fashion. Robotic PCI was successful in 162 out of 164 patients, and operator radiation exposure was dramatically reduced by 92.5%.^40^ The trial demonstrated the safety and feasibility of robotic PCI but was limited mainly to simple-to-moderate coronary disease, excluding highly calcified lesions and primary PCI.

The CORA-PCI (Complex Robotically Assisted Percutaneous Coronary Intervention) evaluated the role of robotic PCI as part of a post-market registry in complex lesions.^41^ Type C lesions represented 69.4% of the cohort; 81.5% of cases were completed by use of the robot only, with the remaining 18.5% requiring partial or full manual assistance. Compared to matched patients from the CathPCI registry, who underwent manual interventions, there was no difference in terms of in-hospital and 12-month major adverse cardiac events.

The second-generation CoroPath GRX adds control over the guiding catheter, thus enhancing the operator’s ability to achieve a higher support level when necessary (which is often the case in neuro-interventions due to the high tortuosity of the cerebral circulation). Initial experience, with the second-generation robot, is positive but needs to be confirmed in future studies.^42^

After proving feasibility and radiation reduction, the next level, and probably one of the most exciting and innovative applications of robotic vascular interventions, is the ability to perform procedures from a remote location.

In a first case series published in 2019, Dr Madder used the CoroPath GRX robot, 20 miles away from the angiography suite, to successfully perform five “tele-stenting procedures” of type A lesions.^43^ All five cases achieved good angiographic results with no peri-procedural complications. This study is a milestone for future remote robotic vascular interventions and opens the road for future interventions in remote areas where no interventionalist is available, including for stroke.

In November 2019, Corindus announced completion of the first-in-human robotic-assisted brain aneurysm embolization using the CoroPath GRX System, performed by Dr Vitor Mendes Pereira from Toronto Western Hospital.^42^ Corindus is now planning the CoroPath GRX neuro-study to evaluate the effectiveness and safety of robotic-assisted endovascular brain embolization procedures.^44^

Nevertheless, full remote thrombectomy is not ready for general use. The present robotic generation still requires a manual phase during vascular access and catheter navigation up to the target vessel.

Contrary to coronary procedures, neurointervention often requires a triaxial approach (guiding catheter, distal access catheter, and microcatheter), which CoroPath GRX cannot handle yet. Tactile feedback, which is crucial to prevent excessive pressure overload and risk for vascular complications, is not available within the present version. Additionally, during a fully remote procedure, in case of complications or technical difficulties, there is no on-site physician able to take over and convert to a classic manual mode, which presents the potential for serious adverse events.

Nevertheless, remote stroke interventions are one of Siemens’s main priorities. Future device generations are already under development, and hopefully the future will see development of robotic systems as safe and efficient as manual procedures, allowing patient treatment at strategically equipped remote locations.

## FINAL THOUGHTS AND CONCLUSION

The tools described in this article and many others are paving the way to helping more stroke patients and achieving better results. Nevertheless, it must be remembered that MT is but one part of the chain in stroke treatment. Stroke patient outcomes are highly dependent upon efficient hyper-acute management, but equally important is their management in the post-acute phase—first in the neurosurgical intensive care unit, then on the neurology ward, and eventually during rehabilitation and re-integration into the community. Medical authorities, when planning stroke programs and strategies, should focus their means on both aspects, to achieve the best outcomes for their stroke patients.

## Abbreviations

AHA: American Heart Association
ASPECTS: Alberta Stroke Program Early CT Score
CSC: comprehensive stroke center
CT: computed tomography
CTA: computed tomography angiography
DWI: diffusionweighted imaging
ENACT: Evaluating Neuroprotection in Aneurysm Coiling Therapy
ESO: European Stroke Organisation
FRONTIER: Field Randomization of NA-1 Therapy in Early Responders
GPS: global positioning system
IVtPa: intravenous thrombolysis
LVO: large-vessel occlusion
MCA: middle cerebral artery
MCAO: MCA occlusion
MT: mechanical thrombectomy
NHPSS: non-human primate stroke scale
NIHSS: National Institutes of Health Stroke Scale/Score
NMDAr: NMDA receptors
nNOS: neuronal nitric oxide synthase
PCI: primary coronary intervention
PSC: primary stroke center
RACE: Rapid Arterial oCclusion Evaluation
STAIR: Stroke Treatment Academic Industry Roundtable
USA: United States of America
